# Practitioner's Bookshelf

**Published:** 1892-02-06

**Authors:** 


### PRACTITIONERS' BOOKSHELF.

### PAPERS ON PHYSICAL EDUCATION.*

During the last twelve months an unostentatious little periodical has made its début; it styles itself "Physique," appears monthly, and forms a series of notes and papers culled from the leading American, continental, and English journals, as well as from societies' transactions which deal with the subject of physical education. The editor explains the *raison d'être* of this publication in the first number, and his justification of its appearance is as follows: "We possess some good books on games and sports, and two or three manuals of school hygiene, but personal hygiene, which deals with the individual, and his habit of life, food, sleep, bathing, clothing, exercise, and the like, has not received as much attention as it deserves, and we shall endeavour to supply the deficiency." With the completion of the first volume the editor is to be congratulated, on the success which has attended his first attempt to fill up this deplorable hiatus in our literature. In these days of musical drill and consequent demands from board-school teachers for pianos for its accomplishment, the grumbles of rate-payers find some justification in the following extract from Nils Posse's paper on Swedish gymnastics: "The Swedish method disapproves utterly of the use of music, for the very simple reason that but few gymnastic movements are rythmical, and cannot be made to be so without sacrificing the movement." No one who has once been a witness of a musical drill, as executed by our volunteers, or by a well-trained troup of London School Board children, can possibly agree with the literal acceptance of Nils Posse's dictum. In England, at least, we have found an exercise which at once combines the graces of Terpsichore with the charms of Orpheus and Pan. Other papers which will interest and instruct the reader are "Physical education and the future of the race," "Relation of physical to mental training," "Over-crowding and model dwellings."

*Collected and Edited by C. Roberts, F.R.C.S. (George Bell & Sons.)

